# In Vivo Identification of H3K9me2/H3K79me3 as an Epigenetic Barrier to Carcinogenesis

**DOI:** 10.3390/ijms241512158

**Published:** 2023-07-29

**Authors:** Maria Cristina Piro, Valeria Gasperi, Alessandro De Stefano, Lucia Anemona, Claudio Raffaele Cenciarelli, Manuela Montanaro, Alessandro Mauriello, Maria Valeria Catani, Alessandro Terrinoni, Alessandra Gambacurta

**Affiliations:** 1 Department of Experimental Medicine, Tor Vergata University of Rome, 00133 Rome, Italy; piro@med.uniroma2.it (M.C.P.); gasperi@med.uniroma2.it (V.G.); alexdest.ads@gmail.com (A.D.S.); anemona@uniroma2.it (L.A.); claudioraffaele.cenciarelli@students.uniroma2.eu (C.R.C.); alessandro.mauriello@uniroma2.it (A.M.); alessandro.terrinoni@uniroma2.it (A.T.); 2 Department of Biomedicine and Prevention, Tor Vergata University of Rome, 00133 Rome, Italy; manuelamontanaro1991@gmail.com; 3 NAST Centre (Nanoscience & Nanotechnology & Innovative Instrumentation), Tor Vergata University of Rome, 00133 Rome, Italy

**Keywords:** epigenetics, chromatin, histone modifier enzymes, H3 methylation, cell reprogramming, cancer, epimarkers

## Abstract

The highly dynamic nature of chromatin’s structure, due to the epigenetic alterations of histones and DNA, controls cellular plasticity and allows the rewiring of the epigenetic landscape required for either cell differentiation or cell (re)programming. To dissect the epigenetic switch enabling the programming of a cancer cell, we carried out wide genome analysis of Histone 3 (H3) modifications during osteogenic differentiation of SH-SY5Y neuroblastoma cells. The most significant modifications concerned H3K27me2/3, H3K9me2, H3K79me1/2, and H3K4me1 that specify the process of healthy adult stem cell differentiation. Next, we translated these findings in vivo, assessing H3K27, H3K9, and H3K79 methylation states in biopsies derived from patients affected by basalioma, head and neck carcinoma, and bladder tumors. Interestingly, we found a drastic decrease in H3K9me2 and H3K79me3 in cancer specimens with respect to their healthy counterparts and also a positive correlation between these two epigenetic flags in all three tumors. Therefore, we suggest that elevated global levels of H3K9me2 and H3K79me3, present in normal differentiated cells but lost in malignancy, may reflect an important epigenetic barrier to tumorigenesis. This suggestion is further corroborated, at least in part, by the deranged expression of the most relevant H3 modifier enzymes, as revealed by bioinformatic analysis. Overall, our study indicates that the simultaneous occurrence of H3K9me2 and H3K79me3 is fundamental to ensure the integrity of differentiated tissues and, thus, their combined evaluation may represent a novel diagnostic marker and potential therapeutic target.

## 1. Introduction

Chromatin’s structure and function are affected by epigenetic changes, such as histone chemical modifications (e.g., acetylation, methylation), nucleosome remodeling, DNA methylation, and non-coding RNA-dependent regulation. Due to their reversibility, all these events confer plasticity to chromatin accessibility and gene expression [1]. Such plasticity is fundamental for embryonic stem cells, as it enables a specific epigenetic cell program, which is acquired in a progressive manner, in lineage commitment during embryogenesis [2,3]. Currently, it is possible to in vitro drive stem cells toward a more differentiated state [4,5], as well as to revert differentiated cells to pluripotency (the so-called “epigenetic reprogramming”) [6,7,8].

Either cell differentiation or reprogramming are associated with several well-studied histone modifications involving histone 3 lysine (H3K) and which are mediated by specific histone-modifying enzymes (HMEs). Among all H3K modifications, H3K methylation represents a crucial determinant that, through different degrees of methylation states, is able to regulate the expression level of related genes. Generally, H3K4me3, H3K36me3, and H3K79me2/3 are associated with active transcription, while H3K27me3 and H3K9me2/3 are associated with repressed genes [9,10,11,12]. A wide global increase in H3K27me3 and H3K4me3 levels occurs, for instance, at the beginning of stem cell differentiation, when chromatin is remodeled to silence genes responsible for stemness and to open lineage-specific genes [13]. Notably, low levels of both H3K79me2/3 and H3K9me2/3 favor the reprogramming of somatic cells, while high levels of these histone modifications represent an epigenetic barrier to dedifferentiation [14,15,16,17]. In this context, a crucial role is played by HMEs, involved in deposing and/or removing methyl groups, thus regulating histone methylation degree [15].

Modifications of the epigenetic landscape also act as drivers of a wide spectrum of diseases, including human cancer [18,19]. During tumorigenesis, cells gradually lose their differentiated phenotype and acquire stem-cell features [20,21,22]. Indeed, the alteration in HME expression and dysregulation of a wide genome distribution of histone modifications and DNA methylation have been observed in several cancer types [23,24,25]. For instance, a significant decrease in H3K79me3 levels has been described in lung, bladder, and skin tumors, when compared with normal tissues [26], and low H3K9me2/3 and H3K4me1 levels have been reported in localized prostate cancer with respect to normal tissue [27]. Therefore, an intimate epigenetic link between healthy adult stem cells and tumors can be assessed. Accordingly, we have previously demonstrated that both blood-derived stem cells (BDSCs) and human neuroblastoma SH-SY5Y cells are able to differentiate into osteoblasts [28,29]. During this differentiation process, BDSCs clearly undergo specific epigenetic changes, involving H3 [9], but no information on epigenetics is available for SH-SY5Y cells.

In this paper, we performed a wide genome analysis of H3 modifications during SH-SY5Y cancer cell differentiation that might allow us to identify some epigenetic signatures involved in this process. We found that the most significant modifications concerned H3 methylation states, involved in chromatin remodeling and representing an in vitro barrier to reprogramming, such as H3K9, H3K27, and H3K79. By extending our study to primary tumor tissues from patients affected by basalioma, and bladder and head and neck carcinomas, we found a cancer-specific expression of H3K27me3 and a positive correlation between H3K9me2 and H3K79me3, with a methylation state in cancer specimens lower than their healthy counterparts. Overall, our results demonstrate that the concomitant high levels of H3K9me2 and H3K79me3 are an epigenetic barrier to carcinogenesis and that the combination of these two H3 marks, rather than one single histone modification, might be useful for cancer diagnosis.

## 2. Results

### 2.1. Wide-Genome Analysis of H3 Marks Identifies Epigenetic Signatures during In Vitro Differentiation and Mirrors DNA Methylation Levels

**Figure 1:**
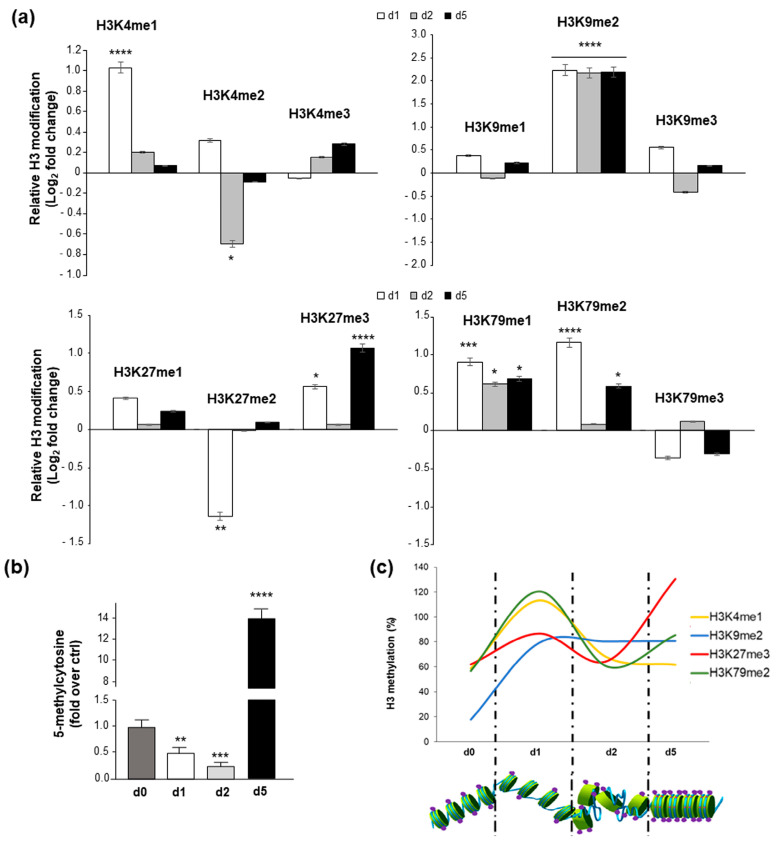
Epigenetic modifications during osteogenic differentiation of human neuroblastoma SH-SY5Y cells. (**a**) Genome-wide analysis of specific H3 modifications (H3K4me1/2/3, H3K9me1/2/3, H3K27me1/2/3, H3K79me1/2/3) in SH-SY5Y cells grown in the presence of 5 µM rapamycin and Geistlich Bio-Oss for 1, 2, and 5 days. Histone marker modifications are expressed as Log2 fold change of H3 methylation over undifferentiated cells. (**b**) DNA methylation after 0, 1, 2, and 5 days of osteogenic differentiation. The amount of 5-methylcitosine is expressed as fold over undifferentiated (day 0) cells. (**c**) Graphical representation of overlapping relationship between H3 methylation changes (H3K4me1, H3K9me2, H3K27me3, H3K79me2) and global DNA methylation state, during differentiation. Results are shown as mean ± S.E.M. of three independent experiments, each performed in triplicate. * *p* < 0.05, ** *p* < 0.01, *** *p* < 0.001, and **** *p* < 0.0001 versus undifferentiated cells.

We have previously demonstrated the ability of human neuroblastoma SH-SY5Y cells to differentiate directly into osteoblasts, already after 5 days [28]. To evaluate the epigenetic impact on this process, the genome-wide distribution of specific H3 modifications (including fifteen different H3 methylation states, four H3 acetylation, and two H3 phosphorylation) at 1, 2, and 5 days was determined [9]. As shown in Figure 1a and Figure S1, differentiation was accompanied by changes in histone H3 modifications, including key lysine residues, generally involved, during differentiation, in transcriptionally active (i.e., H3K4, H3K36, and H3K79) and repressed (i.e., H3K9 and H3K27) chromatin states. As revealed by an ELISA assay, at day 1, we found a significant upregulation of H3K4me1, H3K79me1/2, H3K9me2, and HeK27me3 (Figure 1a and Figure S1b) methylation, as well as of H3K36me3 methylation, H3K56 acetylation, and H3S28 phosphorylation (Figure S1), while there was a significant downregulation of H3K27me2 levels (Figure 1a and Figure S1b). High levels of H3K9me2, H3K79me1, and H3K56ac were persistent up to day 5, when the differentiation process was well established (Figure 1a and Figure S1). Conversely, H3K27me3 and H3K79me2, after being downregulated at day 2, returned to high levels at day 5 (Figure 1a and Figure S1b). Our results demonstrate that cancer cell osteogenic differentiation is characterized by specific H3 mark changes. Some lysine modifications, such as those involving K27 and K9, are closely related to the methylation state of DNA. Therefore, we tried to correlate wide-genome H3 modification data with the cell chromatin state, by analyzing global DNA methylation during differentiation. When compared to control cells (day 0), 5-methylcytosine levels dropped out within 2 days of osteogenic differentiation, and then markedly increased by day 5 (Figure 1b). Notably, DNA methylation changes strongly mirrored the H3 modifications described above (Figure 1c). Collectively, these data point out that the process of chromatin opening/remodeling (as evidenced by increased levels of H3K4me1 and H3K79me1/2; Figure 1c) and its consequent increase in transcriptional activity (as evidenced by increased levels of H3K36me3, H3K56ac, and H3S28P; Figure S1c) begin and also end on day 1 of differentiation.

### 2.2. Immunofluorescence Analysis Identifies H3K9me2 and H3K79me3 as Specific Hallmarks in Human Cancers

**Figure 2:**
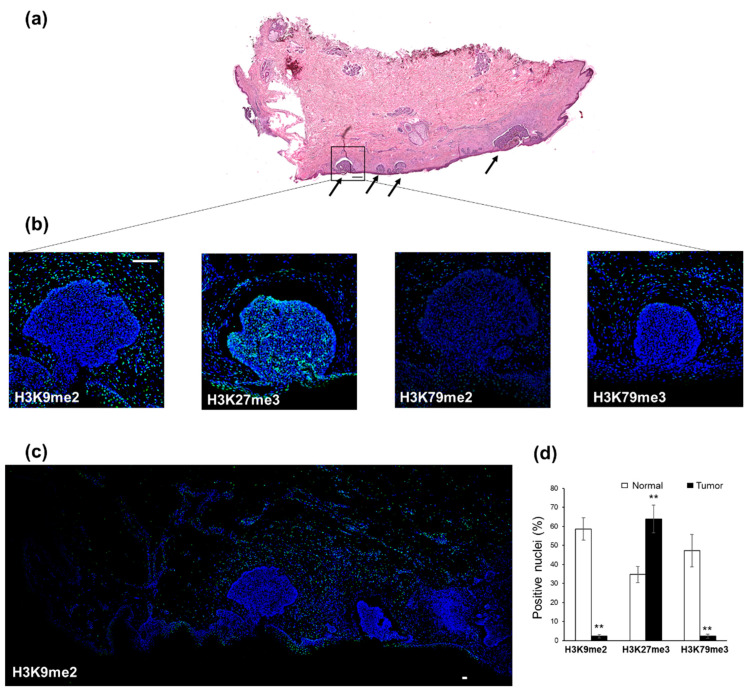
Immunofluorescence analysis of H3 modifications in basalioma. (**a**) Hematoxylin and eosin staining of biopsy showing basalioma (indicated by black arrows) and surrounding normal tissue (4× magnification). Scale bar: 100 µm. (**b**) Immunostaining of human basalioma biopsies with specific FITC-conjugated antibodies directed against H3K9me2, H3K27me3, H3K79me2, and H3K79me3. Nuclei were counterstained with DAPI. Each merged image is representative of eight analyzed samples. 20× magnification. Scale bar: 100 µm. (**c**) Immunostaining with FITC-labeled anti-H3K9me2 antibody. The 4× magnification allows a global view of the entire biopsy. Scale bar: 100 µm. (**d**) Quantitative analysis of H3K9me2-, H3K27me3-, and H3K79me3-positive nuclei, as derived from immunofluorescence images and calculated by ImageJ/Fiji software 2.9.0. Data are the mean ± S.E.M. and reported as percentage of positive nuclei. ** *p* = 0.0078 versus normal counterpart.

We wondered if our in vitro results might be useful to better understand the molecular mechanisms underlying in vivo oncogenesis, as well as to identify potential candidate epigenetic alterations responsible for and/or crucial for maintenance of a normal phenotype. We analyzed, by immunofluorescence, skin biopsy specimens from eight patients, who received a diagnosis of basal-cell carcinoma (basalioma), the most common form of non-melanoma skin cancers [30], which easily allows tumor cells to be distinguished from healthy cells in the same tissue sample (Figure 2a). We focused on three H3 marks, i.e., H3K9me2, H3K27me3, and H3K79me2, which changed significantly during in vitro differentiation.

We found clear differences in the methylation state of each selected marker between tumor regions and normal counterparts (Figure 2 and Figure S2). H3K9me2 was present in all healthy samples, while low expression was noticed in tumor samples (Figure 2b,c); a statistically significant difference in the percentage of positive nuclei was found by quantitative analysis of immunofluorescence data (58.6% and 2.3% in normal and tumor, respectively; *p* = 0.0078) (Figure 2d). On the contrary, the expression of H3K27me3 in cancer regions was significantly higher than that in surrounding normal regions (Figure 2b) (34.8% and 63.9 in normal and tumor, respectively; *p* = 0.0078) (Figure 2d). Although we detected more H3K79me2 in normal regions compared with tumor areas, only few nuclei displayed this enrichment (Figure 2b), in contrast to what we expected. Since the downregulation of H3K79me3 levels has been observed in a variety of cancers [26], we also evaluated this marker in our biopsies. Notably, the trimethylated H3K79 signal was stronger than the dimethylated H3K79 signal in all normal regions surrounding tumor areas, where, conversely, it was markedly lower (Figure 2b). Like H3K9me2, the percentage of positive nuclei was significantly different between normal and tumor regions (47.3% and 2.3%, respectively; *p* = 0.0078) (Figure 2d).

To expand our analysis, we evaluated the same H3 mark profiles in head and neck tumor and urothelial bladder carcinoma, two other common types of epithelial cancer [30]. We analyzed seven non-metastatic head and neck carcinomas and ten non-metastatic bladder cancer biopsies, comparing the methylation status of selected H3 lysine residues with their normal counterparts, fixed on the same slide. 

**Figure 3:**
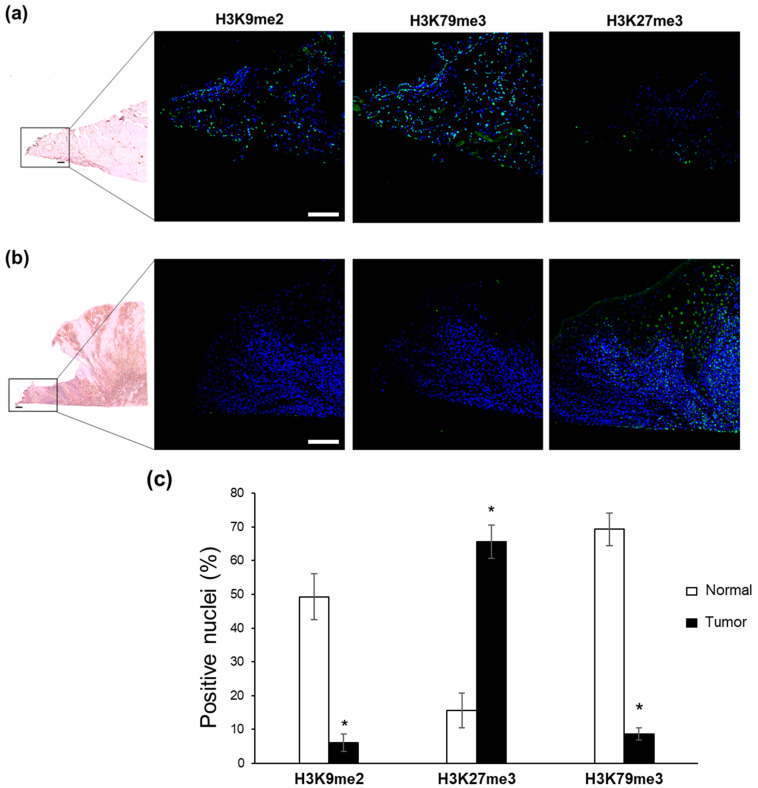
Immunofluorescence analysis of H3 modifications in head and neck cancer. Hematoxylin and eosin staining of biopsies showing (**a**) normal and (**b**) tumor tissues (4× magnification). The 20× magnification shows immunostaining with specific FITC-conjugated antibodies directed against H3K9me2, H3K27me3, and H3K79me3. Nuclei were counterstained with DAPI. Each merged image is representative of seven analyzed samples. Scale bar: 100 µm. (**c**) Quantitative analysis of H3K9me2-, H3K27me3-, and H3K79me3-positive nuclei, as derived from immunofluorescence images and calculated by ImageJ/Fiji software. Data are the mean ± S.E.M. and reported as percentage of positive nuclei. * *p* = 0.016 versus normal counterpart.

The differential levels of H3 methylation in head and neck cancer samples reflected those observed for basalioma (Figure 3 and Figure S3). Both dimethyl H3K9 and trimethyl H3K79 fluorescent signals were detected in the nuclei of non-tumor head and neck epithelium (Figure 3a), but not in the nuclei of tumor counterparts (Figure 3b); the percentage of positive nuclei was 49.3% and 6.8% (for H3K9me2; *p* = 0.016), and 69.3% and 4.9% (for H3K79me3; *p* = 0.016), in normal and tumor regions, respectively (Figure 3c). On the contrary, increased H3K27 trimethylation was evident in all tumor biopsies examined, compared with that observed in related normal specimens (Figure 3a,b), where we found 15.6% of positive nuclei versus 65.6% in tumor ones (*p* = 0.016).

**Figure 4:**
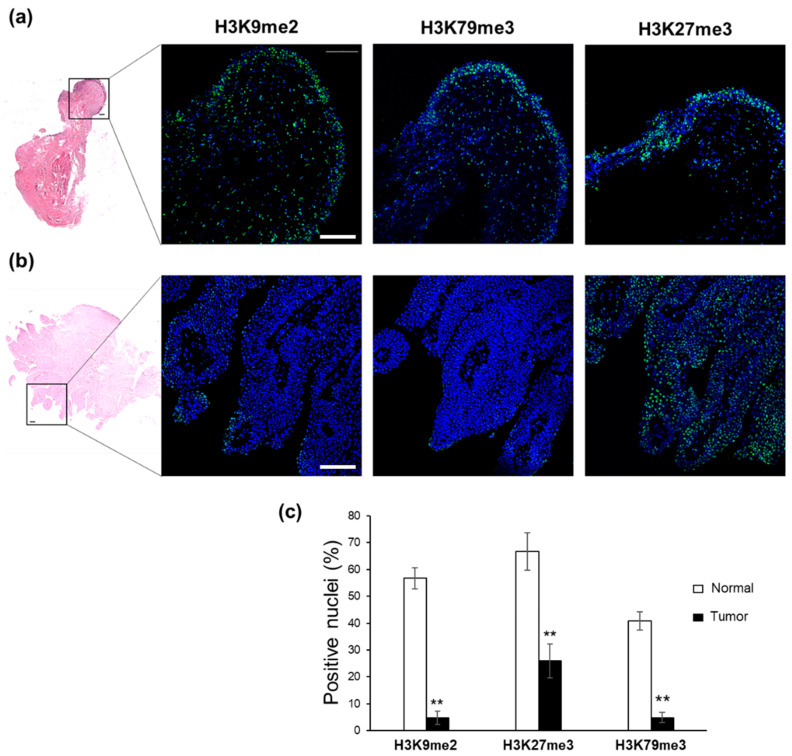
Immunofluorescence analysis of H3 modifications in bladder cancer. Hematoxylin and eosin staining of biopsies showing (**a**) normal bladder and (**b**) tumor counterpart (4× magnification). The 20× magnification shows immunostaining with specific FITC-conjugated antibodies directed against H3K9me2, H3K27me3, and H3K79me3. Nuclei were counterstained with DAPI. Each merged image is representative of ten analyzed samples. Scale bar: 100 µm. (**c**) Quantitative analysis of H3K9me2-, H3K27me3-, and H3K79me3-positive nuclei, as derived from immunofluorescence images and calculated by ImageJ/Fiji software. Data are the mean ± S.E.M. and reported as percentage of positive nuclei. ** *p* = 0.002 versus normal counterpart.

Similar results were obtained in bladder biopsies (Figure 4 and Figure S4), where H3K9me2 and H3K79me3 staining was higher in normal bladder samples (Figure 4a) than in their cancer counterparts, where only a few positive nuclei were detected (Figure 4b); the percentage of positive nuclei was 56.7% and 4.8% (for H3K9me2; *p* = 0.002), and 40.7% and 4.9% (for H3K79me3; *p* = 0.002), in normal and tumor biopsies, respectively (Figure 4c). Regarding H3K27me3, positive green nuclei were detected in both normal and cancer regions (Figure 4a,b); the apparent, more pronounced expression in healthy tissues compared with their related tumor sections (66.7% and 25.9% of positive nuclei, respectively; *p* = 0.002) was, however, not supported by the significant heterogeneity observed among patients (Figure 4c). Overall, our results showed that the low expression of either H3K9me2 or H3K79me3 was markedly associated with tumor phenotype. On the contrary, H3K27me3 expression appeared to be closely related to tumor type. 

### 2.3. H3K9me2 and H3K79me3 Are Positively Correlated in Cancers

**Figure 5:**
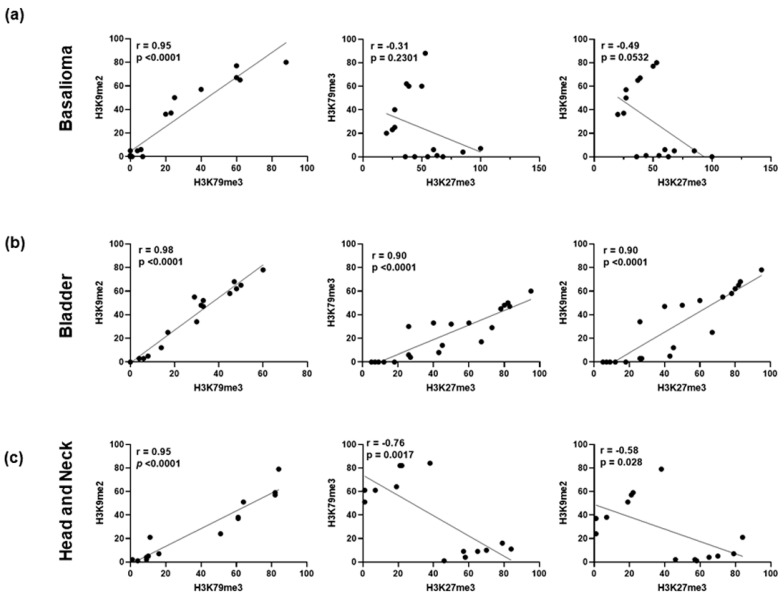
Pearson correlation analysis of different H3 methylation changes. (**a**) Basalioma, (**b**) bladder, and (**c**) head and neck cancers. Pearson correlation coefficient (r) and *p* values are reported in each graph.

To gain further insights, we evaluated whether the above observed H3 modifications might be statistically correlated with each other. Interestingly, we found a significant positive correlation between H3K9me2 and H3K79me3 epigenetic alterations in all examined tissues (*p* < 0.0001), with a Pearson correlation coefficient (r) of 0.95 for both basalioma and head and neck cancer and of 0.98 for bladder cancer (Figure 5). Regarding the relationship of H3K27me3 with the other two H3 marks, results varied depending on the tissue examined. A significant positive correlation was found only in bladder cancer (Figure 5b), where, despite the heterogenous expression found across patients, the H3K27me3 mark overall followed the trend of the other two flags. Conversely, we found no statistical significance for basalioma (absence of correlation) and head and neck tumors (apparent negative correlation) (Figure 5a,c).

Overall, Pearson correlation analysis revealed that the simultaneous low expression of both H3K9me2 and H3K79me3 was closely associated with all examined cancer types, thus suggesting that combinations of these two histone modifications might contribute to distinguish normal from tumor phenotypes.

### 2.4. Bioinformatics Identifies Differentially Expressed Genes Involved in H3K9 and H3K79 Methylation in Cancers 

To better survey biological mechanisms underlying H3 mark differences observed in our samples, we focused on 14 specific histone H3K9- and H3K79-modifying enzymes that might be differentially regulated in primary tumor samples compared to normal counterparts. In particular, we evaluated (i) “writers”, i.e., histone methyltransferases responsible for the dimethylation (namely, EHMT2 (G9a) and EHMT1 (GLP)) and trimethylation (SETDB1, SETDB2, SUV39H1, and SUV39H2) of K9, as well as for the methylation of K79 (DOT1L); (ii) “readers”, i.e., proteins belonging to the PRDM family (such as PRDM1, PRDM5, PRMDM6), involved in the K9 dimethylation complex, as well as ATAD2 responsible for the H3K9me2-to-H3K9me3 transition in heterochromatin [31]; and (iii) “erasers”, i.e., enzymes responsible for H3K9me2 demethylation (i.e., KDM3A, KDM3B, KDM4A). Three online available RNA-seq datasets were downloaded from the NCBI GEO repository: the GSE125285 dataset, containing gene expression profiling of 35 basalioma and their adjacent normal tissues [32]; the GSE133624 dataset, including 36 cases of bladder cancer and 29 adjacent normal bladder tissues [33]; and the GSE112026 dataset, containing 46 oropharyngeal squamous cell carcinoma and 25 normal tissue samples [34]. Notably, only PRDM6 was absent in GSE125285 basalioma dataset.

**Figure 6:**
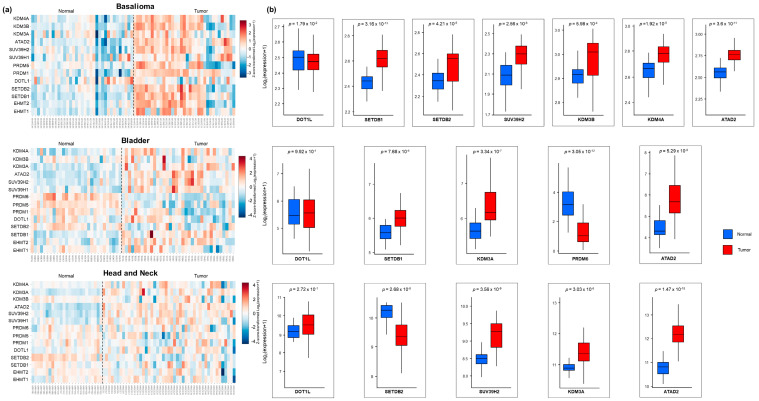
Differential expression gene (DEG) analysis of RNA-seq datasets of normal and tumor samples. (**a**) Heatmaps illustrating up- and down-regulated K9/K79 modifier enzymes in tumor and normal samples. DEGs are reported as Log2 fold change. Blue to red shows the low- to high-gene-expression trend, respectively. (**b**) Differential expression of statistically significant single genes involved in chromatin remodeling. Data are reported as Log2 (expression +1). *p* values are reported in each graph.

As indicated by heatmaps (Figure 6a), several genes encoding “writers”, “readers”, and “erasers” displayed differential expression patterns in all specimens. Neither DOT1L expression nor levels of methyltransferases responsible for K9 dimethylation showed statistically significant changes in all tumor types. Conversely, we found changes in methyltransferases implicated in K9 trimethylation in all tumor specimens: in particular, basalioma showed an upregulation of SETDB1, SETDB2, and SUV39H2; bladder cancer showed an upregulation of SETDB1; and head and neck cancer showed an upregulation of SUV39H2 and downregulation of SETDB2 (Figure 6b). In parallel, an overall increase in H3K9me2 erasers (KDM3B and KDM4A in basalioma, and KDM3A in the other two tumor types) and a bladder-specific decrease in the reader PRDM6 involved in K9 dimethylation were found (Figure 6b). Finally, in all tumors, the most significant upregulated gene was the reader ATAD2 (Figure 6b) involved in the H3K9me2-to-H3K9me3 transition [31]. This last finding could explain the downregulation of H3K9me2 in tumor samples and suggest a potential shift toward trimethylation.

## 3. Discussion

Epigenetics plays a crucial role in gene expression related to cell differentiation or reprogramming, and some epigenetic signatures are shared by stem and tumor cells, both characterized by an undifferentiated phenotype. Here, we have in vivo identified specific epigenetic barriers that are commonly knocked down during carcinogenesis, which may have clinical relevance.

Based on our previous data [28,29], we first explored whole-genome epigenetic changes in an in vitro model of tumor programming, demonstrating that reversal of the tumoral phenotype is associated with chromatin opening/remodeling (allowed by increased H3K4me1 and H3K79me1/2 levels) and its consequent gain in transcriptional activity (due to increased H3K36me3, H3K56ac, and H3S28P levels, as well as to the decreased 5-methylcytosin content). Notably, all of these are early changes, already occurring in the first day of differentiation (Figure 1 and Figure S1). In particular, increased H3K4 monomethylation accounts for enhanced activation during differentiation, promoting chromatin interactions between distal enhancers and promoters [35]. In parallel, the drastic decrease in the global H3K27me2/me3 ratio is required for chromatin reconfiguration: this ratio is a recently recognized dynamic parameter defining the cell-lineage-specific transcriptional program or pluripotency maintenance. During differentiation, the epigenetic switch allows H3K27me2 flags to be resolved into either H3K27 acetylation or H3K27me3, as a response to gene expression requirements [36]. Notably, acquisition of the differentiated phenotype requires the restoration of H3K9me2 and H3K79me2 epigenetic barriers to reprogramming (Figure 1 and Figure S1) [14,15,16,17]. 

These in vitro findings prompted us to explore whether some of these H3K marks might be associated with the maintenance of a healthy differentiated state, with implications in oncogenesis. Since knowledge of H3K9, H3K27, and H3K79 methylation states on the tumors/normal tissue of patients is still elusive, we tested it in ex vivo sections of three epithelial tumors (basalioma, bladder and head and neck carcinomas). Our immunofluorescence analysis revealed a global downregulation of H3K9me2 levels in all tumor specimens compared to their healthy counterparts, thus confirming the relevance of H3K9me2 loss in epigenetic barrier breakdown that occurs during carcinogenesis. Most likely, this loss is due to the transition of the methylation state of H3K9me2 to demethylation and/or trimethylation originating from the upregulation of KDM3A, KDM3B, and KDM4A demethylases, as well as of SETDB1, SETDB2, and SUV39H2 trimethylases (Figure 6). In this context, the ATAD2 upregulation observed in all tumors may contribute to the H3K9me2-to-H3K9me3 transition, as recently demonstrated in *Schizosaccharomyces pombe*, a yeast model displaying heterochromatin organization similar to human cells [31]. Whatever the mechanism leading to H3K9me2 depletion, our data highlight the in vivo role of H3K9me2 in sustaining differentiated cell type identity and counteracting oncogenic transformation [37]. H3K9me2 represents an evolutionarily conserved histone modification, specific to nuclear peripheral heterochromatin, which is known to be required for cell division [38] but, importantly, needed for chromosomal (3D) conformational changes able to generate heterochromatic compartments in eukaryotic cells [39]. Genome regions containing these epigenetic modifications are, indeed, transcriptionally repressed by switching from A (active) to B (inactive) compartments [39]. Accordingly, the function of H3K9me2 as a barrier to reprogramming is explained by its ability to block DNA binding of the pluripotency transcription factors OCT4, SOX2, Krüppel-like factor 4 (KLF4), and MYC [38,40], thus preventing inappropriate gene expression [41].

On the basis of our results, we also indicate the higher methylation state of H3 lysine 79 (H3K79me3) as another distinct epigenetic signature that allows for distinguishing healthy from tumor tissues. Unlike our in vitro findings (Figure 1) and literature data [14,15,16,17], H3K79me2 levels did not change substantially in vivo, while H3K79me3 content, found elevated in healthy tissues, drastically decreased in tumor counterparts (Figure 2, Figure 3 and Figure 4). Why should levels of H3K79 trimethylation be lower in tumor cells than in healthy, differentiated cells? It is well known that H3K79 methylation is involved in RNA-polymerase-II-mediated transcription, DNA repair, genomic stability, and cell-cycle checkpoints [42,43], as well as in cell fate determination and maintenance of terminal differentiation [44]. Recently, Chory and co-workers demonstrated that the methylation kinetics of H3K79 (me1/me2/me3) is strictly dependent on the nucleosome turnover rate. Indeed, unlike H3K27 and H3K4, whose complete trimethylation occurs in 10–20 min, H3K79 trimethylation requires a minimum of 72 h [45]. Therefore, we hypothesize that the lower nucleosome turnover rate, the higher the number of H3K79me3 flags deposed; the global decrease in H3K79me3 levels observed in basalioma, and bladder and head and neck carcinoma specimens can mirror the increase in cell proliferation and nucleosome turnover rate, typical of stem and tumor cells. Notably, no H3K79 demethylase has been identified up to now and the DOT1L (H3K79 methyltransferase) expression was similar in healthy and tumor cells (Figure 6), thus confirming the inverse relationship between H3K79 trimethylation state and nucleosome turnover rate due to fast cell division.

Unlike H3K9me2 and H3K79me3, our data on H3K27me3 mark did not allow us to draw univocal conclusions. Indeed, H3K27me3 expression appeared to be strictly related to tumor type, as we found low levels in all basalioma and head and neck tumor samples, but not in bladder cancer specimens, where, conversely, we observed an increase (although with significant heterogeneity across patients) compared with their matched normal tissues. Other studies have shown apparently conflicting data on H3K27me3 expression, as higher levels were found in primary prostate cancer cells, in comparison with normal cells [46], while a lower expression was described in endometrial cancer [47]. Incidentally, both oncogenic and tumor suppressive functions have been reported for the H3K27me3 writer, EZH2 [48,49,50]. Therefore, due to its dichotomous behavior, H3K27me3 cannot be considered as an epigenetic barrier.

Collectively, our data provide further insights into the role of H3K9me2 and H3K79me3 in cancer epigenetics. Despite the relatively small number of samples, attractive conclusions can be drawn from our uniform and univocal results, and important strengths of our study can be underlined. To the best of our knowledge, this is the first in vivo study showing wide-genome analysis of H3K9me2 and H3K79me3 epigenetic signatures in cancer. Evanno and co-workers described decreased H3K79me3 content in lung, bladder, and skin tumors, in comparison with normal tissues [26]; Ellinger’s group reported low H3K9me2 levels in localized prostate cancer with respect to normal tissue [27]. However, H3K9me2 and H3K79me3 have never been evaluated in combination. The novelty of this investigation is the finding of a strong positive correlation between these two epigenetic modifications (Figure 5), thus indicating that the simultaneous occurrence of these two obstacles to dedifferentiation ensures the integrity of differentiated tissues and prevents cancer transformation. Lastly, these findings have strong relevance, taking into account that, unlike available literature data, they were obtained by analyzing patient-matched tumors and normal tissues, thus reducing the inter-individual variability of epigenetic background [51,52].

In conclusion, the H3K9me2/H3K79me3 combination represents a valuable diagnostic marker and potential therapeutic target for restoration of a “normal epigenome”.

## 4. Materials and Methods

### 4.1. Cell Cultures

The human neuroblastoma SH-SY5Y cell line (ATCC CRL-2266) was grown on tissue culture dishes (BD Falcon), in DMEM-F12 with 15 mM HEPES and 2 mM L-glutamine (Lonza), supplemented with 10% (*v*/*v*) Fetal Bovine Serum (FBS) and 1% (*v*/*v*) penicillin (100 U/mL)/streptomycin (100 mg/mL), and maintained in a humidified incubator at 37 °C with 5% (*v*/*v*) CO_2_. At confluence, cells were detached with 1× trypsin solution, centrifuged at 272× *g* for 7 min, and seeded again (2 × 10^6^ cells/well), in complete medium. During the study, cells were regularly tested for mycoplasma absence, by using either MycoStrip™-Mycoplasma Detection Kit (InvivoGen, Toulouse, France) or LookOut^®^ Mycoplasma qPCR Detection Kit (Sigma Aldrich, Sant Luis, MO, USA).

### 4.2. Osteogenic Differentiation

SH-SY5Y cells (3 × 10^4^/well) were seeded into a 24-well plate pre-coated with 0.1 mg/mL of collagen type I rat tail high-concentration solution (BD Pharmigen, San Diego, CA, USA) and incubated at 37 °C for 24 h. To induce osteogenic differentiation, the osteogenic inductor rapamycin (5 µM) (Selleckchem, Boston, MA, USA) and the Geistlich Bio-Oss, a spongious bone substitute of bovine origin scaffold (particle size of 0.25–1.0 mm) (Geistlich Pharma AG, Wolhusen, Switzerland), were added to each well, the day after. Osteogenic differentiation was monitored at 1, 2, and 5 days, with the medium changed every two days. Cells were collected at each time point and used for all experiments. 

### 4.3. Histone H3 Modification Analysis

Total histones were isolated by using EpiQuik Total Histone Extraction Kit (Epigentek, Farmingdale, NY, USA, cod. OP-0006), according to manufacturer instructions. Briefly, cell pellets were resuspended in 1 mL of Diluted Pre-Lysis Buffer (1×) and placed on ice for 10 min under stirring to remove plasma membranes. Pellets, obtained after centrifugation at 9391× *g* for 1 min at 4 °C, were resuspended in Lysis Buffer (50 μL) and incubated on ice for 30 min. Lysates were then centrifuged at 13,523× *g* for 5 min at 4 °C and supernatants transferred to a new tube. To avoid scaffold precipitates hindering histone extraction, 0.3 volumes of Balance Buffer without dithiothreitol (DTT) were immediately added to each sample. Analysis of twenty-one H3 modifications was performed by using the EpiQuik Histone H3 Modification Multiplex Assay Kit (Epigentek, cod. P-3100), according to manufacturer instructions. For each sample, the amount of histone H3 modification was compared to that of total H3 and expressed as Log2 fold change.

### 4.4. Global DNA Methylation Analysis

Global methylation status of genomic DNA isolated from cells was evaluated through a rapid colorimetric assay, by using the MethylFlash Global DNA Methylation (5-mC) ELISA Easy Kit (Epigentek cod. P-1030), which specifically measures 5-methylcytosine levels. For each assay, 0.1 µM genomic DNA was used. The percentage of 5-mC was calculated according to manufacturer instructions. 

### 4.5. Sample Collection

Human biopsies, including tumor and relatively healthy counterparts, were collected from 25 diagnosed cancer patients (15 male and 10 female) admitted at Policlinico Tor Vergata, University of Rome. The median age was 64 for males (age range 42–92 years) and 63 for females (age range 24–88 years). A total of 8 patients received a diagnosis of basalioma, 10 patients were diagnosed for bladder cancer, and 7 for head and neck cancer. Study design was carried out according to the protocol approved by the Ethical Committee of Policlinico Tor Vergata, University of Rome (authorization Nr129/18).

### 4.6. Histological and Immunofluorescence Analysis

Surgical samples were fixed for 24 h in 4% buffered formalin (Bio Optica, Milan, Italy) immediately upon removal. Thus, samples were dehydrated with an Automatic Tissue Processor for histologic specimens (FTP300 processor, Bio Optica) and subjected to diaphanization in xylene (Bio Optica) and impregnation in liquid paraffin (Bio Optica). Serial sections (3 µm) were obtained using a microtome (Microm HM440E, Leyca Biosystems, Deer Park, IL, USA) and collected on polarized superfrost slides (SuperFrost Plus, Menzel-Glaser, VWR International, Radnor, PA, USA). For each specimen, a deparaffinized section stained with hematoxylin–eosin was analyzed using Ventana Image Viewer software (Version 3.1.4), while additional sections were used for immunofluorescence analysis, as follows. Slides were incubated with Blocking Solution (5% Goat Serum) for 60 min and then incubated overnight at 4 °C with the following specific primary antibodies: anti-H3K27me3 (Epigentek, cod. A-4039), anti-H3K79me2 (Epigentek, cod. A-4044), anti-H3K79me3 (Epigentek, cod. A-4045), and anti-H3K9me2 (Epigentek, cod. A-4035). After washing with 0.1% Tween 20 in 1× PBS, slides were incubated for 60 min with goat anti rabbit IgG (Alexa Fluor^®^ 488, Thermofisher, Waltham, MA, USA, cod A-11008) and DAPI (1:1000) and washed with 0.1% Tween 20 in 1x PBS; afterward, coverslips were mounted using Antifade (Fluoroshield™, Merck, Burlington, MA, USA) and left to dry at 4 °C overnight. Image acquisition was performed with Nikon Confocal Microscope A1. ImageJ/Fiji Software [53] was used to calculate the percentage of positively stained nuclei in each immunofluorescence image. Immunofluorescence quantitative data were obtained by measuring the percentage of nuclei co-positive for DAPI and specific fluorescent probe staining.

### 4.7. Bioinformatic Analysis

To evaluate specific H3K9 and H3K79 modifier enzymes, we selected and downloaded three RNA-seq including normal tissue and carcinoma pairs from GEO database. In particular, the GSE125285 dataset contained gene expression profiling of 35 paired basal cell carcinoma [32], the GSE133624 dataset contained 36 cases of urothelial bladder carcinoma and 29 adjacent normal bladder tissues (22 pairs) [33], and the GSE112026 dataset contained 46 HPV-positive oropharyngeal squamous cell carcinoma and 25 normal tissue samples [34].

Differential gene expression (DEG) analysis was performed by using iDEP 1.0 [54] and eVitta online tools [55].

### 4.8. Statistical Analysis

Data are expressed as mean ± S.E.M. Nonparametric two-way analysis of variance (ANOVA), followed by Dunnett’s multiple comparisons test, and Wilcoxon matched pairs signed rank test were applied, where appropriate. Pearson correlation coefficients were calculated to determine associations between H3 modifications. Differences were considered statistically significant at *p* < 0.05. All statistical analysis was performed using GraphPad Prism version 9.4.1 for Windows (GraphPad Software, San Diego, CA, USA).

## Data Availability

Not applicable.

## Supplementary Materials

The supporting information can be downloaded at: https://www.mdpi.com/article/10.3390/ijms241512158/s1.
