# Supplementary material for: In Vivo Identification of H3K9me2/H3K79me3 as an Epigenetic Barrier to Carcinogenesis
Source: Int J Mol Sci. 2023 Jul 29;24(15):12158. doi: 10.3390/ijms241512158 (PMC10419041; doi:10.3390/ijms241512158)
Supplement: Supplementary file 1 [file ijms-24-12158-s001.zip › ijms-2513953-supplementary.pdf]

Supplementary figures

In Vivo Identification of H3K9me2/H3K79me3 as an Epigenetic Barrier to Carcinogenesis

Maria Cristina Piro [1#], Valeria Gasperi [1#], Alessandro De Stefano [1], Lucia Anemona [1], Claudio Raffaele Cenciarelli [1], Manuela Montanaro [2], Alessandro Mauriello [1], Maria Valeria Catani [1*], Alessandro Terrinoni [1] and Alessandra Gambacurta [1,3*]

1 Department of Experimental Medicine, Tor Vergata University of Rome, 00133 Rome, Italy; piro@med.uniroma2.it (C.P.); gasperi@med.uniroma2.it (V.G.); alexdest.ads@gmail.com (A.D.S); anemona@uniroma2.it (L.A.); .claudioraffaele.cenciarelli@students.uniroma2.eu (C.R.C.); alessandro.mauriello@uniroma2.it (A.M.); catani@uniroma2.it (M.V.C.); alessandro.terrinoni@uniroma2.it (A.T.); gambacur@uniroma2.it (A.G.)

2 Department of Biomedicine and Prevention, Tor Vergata University of Rome, 00133 Rome, Italy; manuelamontanaro1991@gmail.com (M.M.)

3 NAST Centre (Nanoscience & Nanotechnology & Innovative Instrumentation), Tor Vergata University of Rome, 00133 Rome, Italy;

* Correspondence: gambacur@uniroma2.it Tel.: +39 06 72596488 (A.G.); catani@uniroma2.it Tel.: +39 06 72596465 (M.V.C.)

# Co-first authors

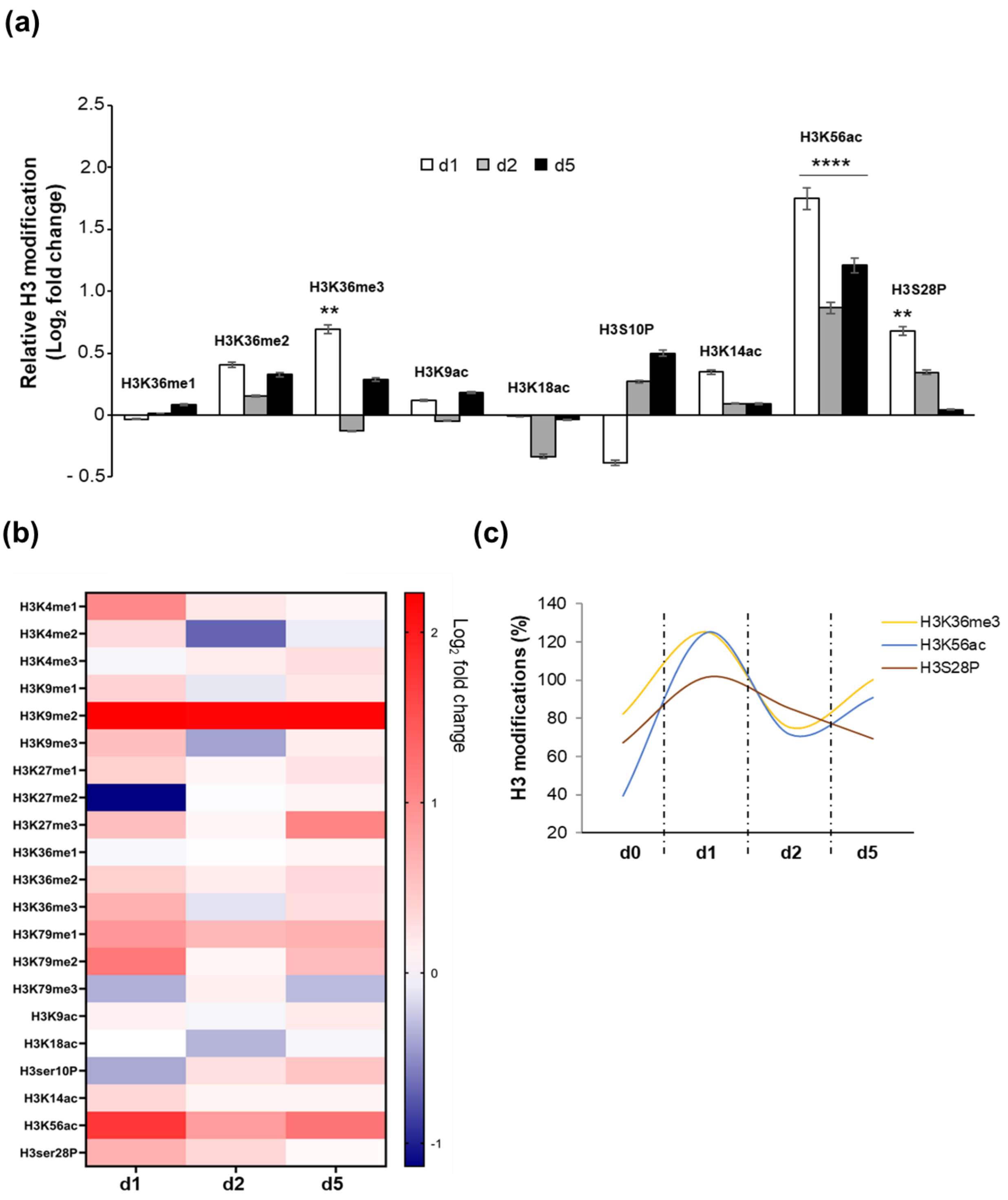

Supplementary Figure S1. Epigenetic modifications during osteogenic differentiation of human neuroblastoma SH-SY5Y cells. (**a**) Genome-wide analysis of specific H3 modifications (H3K36me1/2/3, H3K9ac, H3K18ac, H3S10P, H3K14ac, H3K56ac, H3S28P) in SH-SY5Y cells grown in the presence of 5 μM rapamycin and Geistlich Bio-Oss for 1, 2 and 5 days. Histone marker modifications are expressed as Log2 fold change of H3 methylation over undifferentiated cells. (**b**) Heatmap of changes in histone H3 modifications during differentiation of SH-SY5Y cells treated as in (**a**). Data are expressed as Log2 fold change. (**c**) Graphical representation of statistically significant H3 methylation changes reported in (a). Results are shown as mean ± S.E.M. of three independent experiments, each performed in triplicate. ** $p < 0.01$ and **** $p < 0.0001$ *versus* undifferentiated cells.

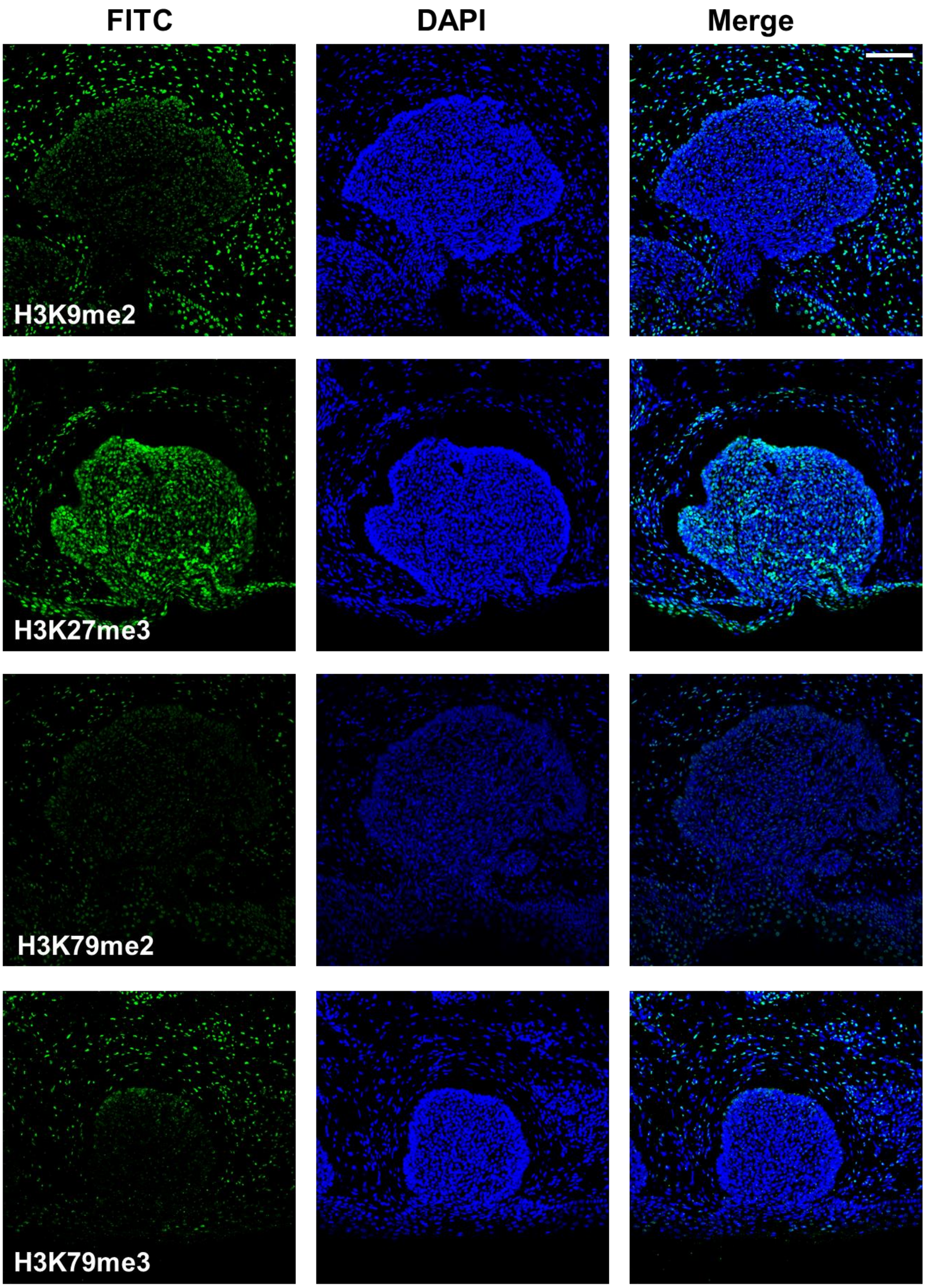

Supplementary Figure S2. Immunofluorescence images of H3 modifications in basalioma biopsy (shown in Figure 2) with FITC channels (green), DAPI channels (blue) and merge of the two fluorescence signals (last panel). 20× magnification. Scale bar: 100 µm.

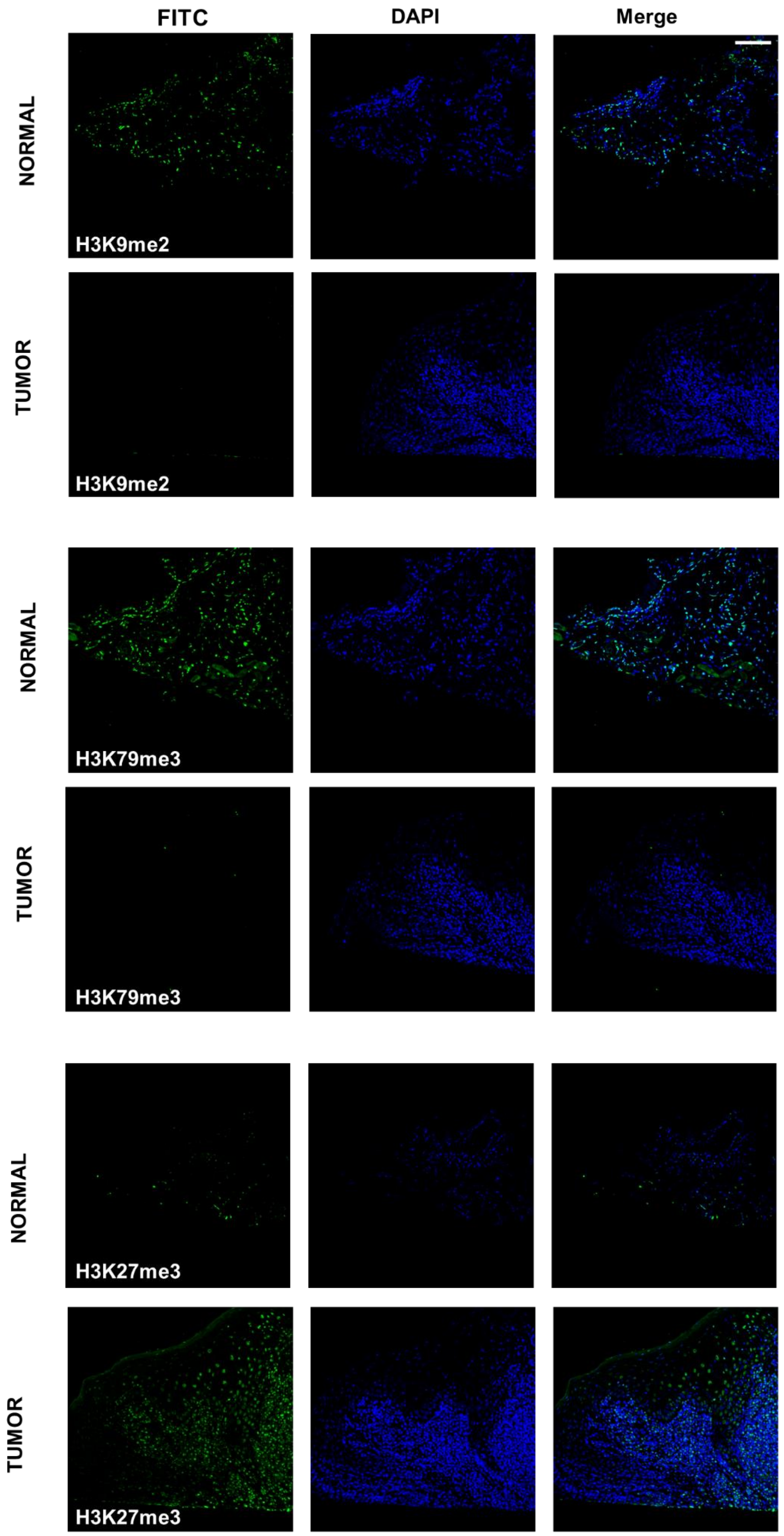

Supplementary Figure S3. Immunofluorescence images of H3 modifications in tumor and normal head and neck biopsy (shown in Figure 3) with FITC channels (green), DAPI channels (blue) and merge of the two fluorescence signals (last panel). 20× magnification. Scale bar: 100 μm.

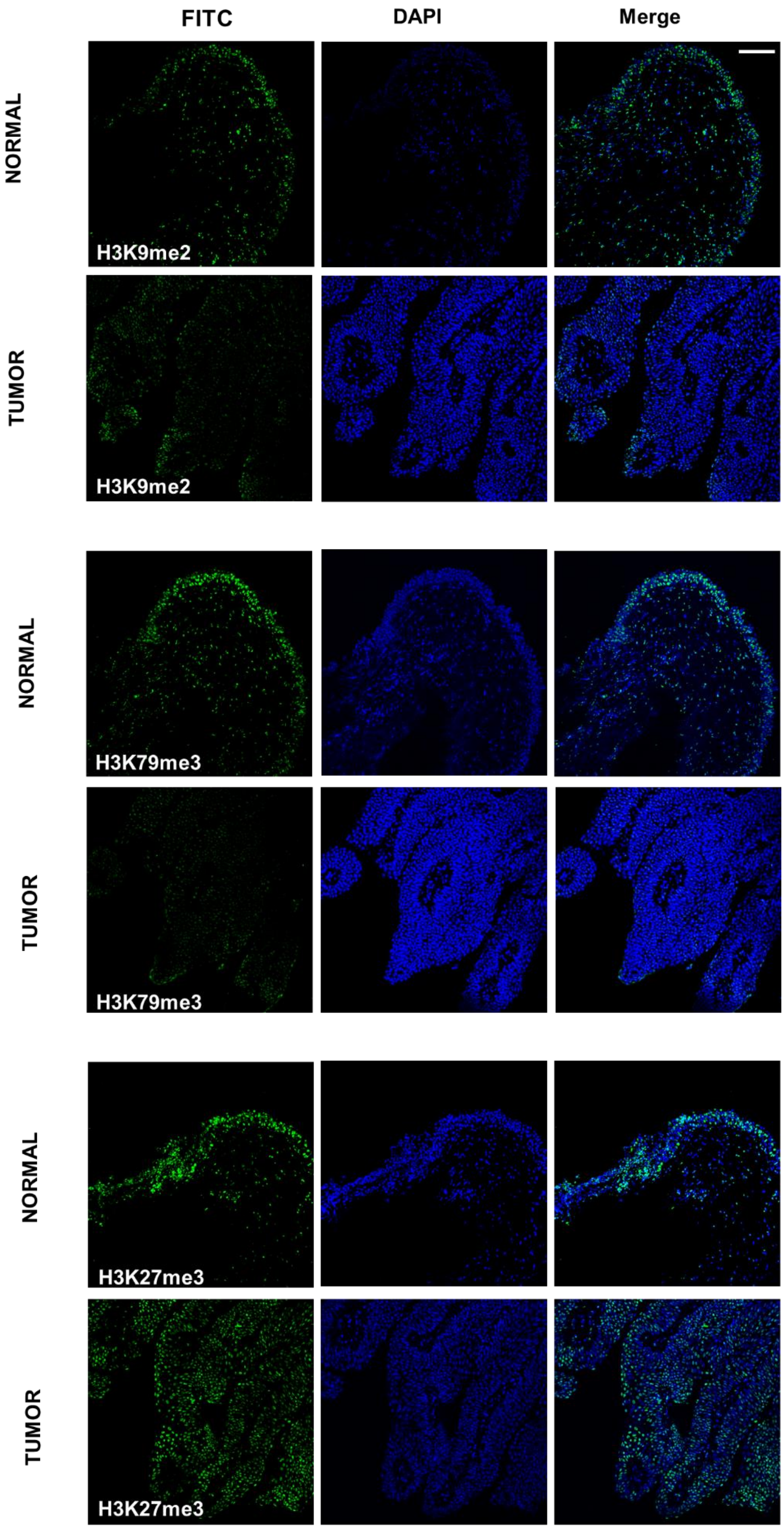

Supplementary Figure S4. Immunofluorescence images of H3 modifications in tumor and normal bladder biopsy (shown in Figure 4) with FITC channels (green), DAPI channels (blue) and merge of the two fluorescence signals (last panel). 20× magnification. Scale bar: 100 μm.
